# FabAV antivenin use after copperhead snakebite: clinically indicated or knee-jerk reaction?

**DOI:** 10.1186/s40409-016-0056-9

**Published:** 2016-01-13

**Authors:** Stephen C. Gale, Jo Ann Peters, LaDonna Allen, Robert Creath, Viktor Y. Dombrovskiy

**Affiliations:** Division of Trauma, Department of Surgery, East Texas Medical Center, Tyler, TX USA; Department of Emergency Medicine, East Texas Medical Center, Tyler, TX USA; Department of Surgery, Rutgers/RWJMS, New Brunswick, NJ USA; Director of Trauma Services, East Texas Medical Center, Level 1 Trauma Center, 1020 E. Idel St., Tyler, TX 75701 USA

**Keywords:** Copperhead, Venomous snakebite, Antivenin, FabAV, Rural

## Abstract

**Background:**

Crotalidae Polyvalent Immune Fab (Ovine) (FabAV) antivenin is commonly recommended after pit viper snakebites. Because copperhead envenomations are usually self-limited, some physicians are reluctant to use this costly treatment routinely, while others follow a more liberal approach. We hypothesized that, in practice, only patients with evidence of significant (moderate or severe) copperhead envenomation [those with snakebite severity score (SSS) > 3] receive FabAV and examined a large cohort to determine the relationship between clinical findings and FabAV administration.

**Methods:**

All data from patients evaluated for copperhead snakebite at a rural tertiary referral center from 5/2002 to 10/2013 were compiled. Demographics, transfer status, antivenin use, and clinical findings were collected; SSS was calculated. The relationships among FabAV use, clinical findings, and SSS were analyzed using *t*-test, chi-square, and Pearson’s coefficient (*p* < 0.05 was significant).

**Results:**

During the study period, 318 patients were treated for copperhead snakebite; 44 (13.8 %) received antivenin. Median dose was four vials (range: 1–10; IQR: 4,6). There were no deaths. Most patients receiving FabAV (63.6 %) were admitted. With regard to demographics and symptoms, only the degree of swelling (moderate vs. none/mild; *p* < 0.01) and bite location (hand/arm vs. leg: *p* < 0.0001) were associated with FabAV use. A SSS > 3, indicating moderate or severe envenomation, was only very weakly correlated with antivenin use (r = 0.217; *p* < 0.0001). The majority of patients with SSS > 3 (65.8 %) did not receive antivenin while most patients who did receive antivenin (70.5 %) had SSS ≤ 3 (indicating mild envenomation).

**Conclusions:**

Considerable variation occurs in antivenin administration after copperhead snakebite. Use of FabAV appears poorly correlated with patients’ symptoms. This practice may expose patients to the risks of antivenin and increasing costs of medical care without improving outcomes. Guidelines used for treating other pit viper strikes, such as rattlesnake or cottonmouth snakebite may be too liberal for copperhead envenomations. Our data suggests that most patients with mild or moderate envenomation appear to do well independent of FabAV use. We suggest, for patients with copperhead snakebite, that consideration be given to withholding FabAV for those without clinical evidence of severe envenomation until prospective randomized data are available.

## Background

Crotalid (pit viper) snakebites are common in the southern United States during the warmer months. In East Texas, and in most other regions of the country, those from copperheads (*Agkistrodon contortrix*) are the most common type of venomous snakebite. While copperhead envenomations are typicallyself-limited, recommendations by poison control centers and toxicologists often do not discriminate between pit viper species in treatment algorithms and commonly recommend polyvalent Crotalinae ovine immune Fab (FabAV) antivenin for most crotalid snakebites (rattlesnake, copperhead, and cottonmouth species) [1–3]. Yet after copperhead snakebite, many physicians are reluctant to use this very costly treatment due to the often self-limited nature of that species’ envenomations. Other clinicians, however, are concerned about disregarding the advice of poison control center experts. As such, we have observed a significant practice variation amongst emergency physicians and surgeons in the region. We hypothesized that copperhead envenomation is typically mild and self-limited and does not require FabAV therapy. We sought to analyze outcomes for a large series of patients struck by copperhead snakes in East Texas with an emphasis on clinical presentation and antivenin usage.

## Methods

Approval was obtained, including waiver of consent, from the East Texas Medical Center (ETMC) institutional review board; emergency department registries from our 450-bed regional tertiary care center were then retrospectively queried for all patients evaluated for acute venomous snakebite from 1/2002 to 12/2013 based on discharge diagnoses and ICD-9-CM codes (E905.0). Only patients with documentation of distinct fang marks were included in the analysis.

Snake species was reported based on patient accounts. As victims are discouraged from hunting and killing venomous snakes for identification, no other reliable method exists for species confirmation. Copperhead species are typically easily distinguished by the coloration, markings, habitat, and other clues from other pit viper species (rattlesnake and cottonmouth). Further, copperhead species are, by far, the most common cause of snakebite in the East Texas region [4].

Demographics, snake species, clinical findings, laboratory data and admission data, and antivenin use were abstracted from the individual electronic charts including those sent during from transferring facilities and scanned into the electronic medical record.

### Severity of envenomation

From the medical record, numbness, bruising, and erythema were reported as present or absent. In contrast, swelling and pain were described by degree – none (0), mild (1), moderate (2), and severe (3) – based on physician or nursing documentation. Coagulopathy was assessed as any of the following: platelet count < 150,000/mm^3^; decrease in platelet count by > 25 %; prothrombin time (PT) > 13.5 s; partial thromboplastin time (PTT) > 40 s; fibrinogen level < 200 mg/dL. A patient was considered to have cardiovascular symptoms if there was any evidence of hemodynamic compromise indicated by any of the following: systolic blood pressure (SBP) < 90 mmHg; diastolic blood pressure (DBP) < 50 mmHg; heart rate (HR) > 130 bpm or increase in HR > 30 bpm during the assessment period. Constitutional symptoms included any of the following: nausea, vomiting, dizziness, fever, chills, or abdominal cramps/pain. The snakebite severity score (SSS), a validated research tool for assessing the severity of envenomation after pit viper snakebite, which is also commonly included in algorithms to determine the need for FabAV was calculated based on physician documentation and available laboratory data [5–7]. The SSS is commonly interpreted or grouped as mild (≤3), moderate (4–7), and severe (≥8).

### Outcomes

Specific outcomes (admission status, length of stay, complications, and mortality) were also compiled and analyzed. Patients receiving FabAV were compared to those who did not for individual clinical symptoms, SSS and outcomes using *t*-test, Mann–Whitney *U* test, and chi-square when appropriate. The degree of envenomation (“mild” versus “moderate to severe”), as indicated by the SSS > 3, was correlated to FabAV administration using Pearson’s correlation. A *p* < 0.05 was considered significant for all statistical tests.

## Results

### Demographics

During the study period, 335 patients were evaluated for venomous snakebite at ETMC; 318 (94.9 %) were known or presumed copperhead snakebites. Of these, 123 (38.7 %) were initially evaluated at smaller hospitals or clinics prior to transfer to ETMC. The age range was 2 to 89 years. Sixty-two patients (19.5 %) were children (age < 18). Demographics are summarized in Table 1. No specific demographic category was associated with FabAV administration.

**Table 1:** Patient demographics

	Total (318)	FabAV (44)	No FabAV (274)	*p*
Age – years (mean ± SD)	37.4 ± 19.6	39.4 ± 18.3	37.2 ± 18.3	NS
Sex – % male	59.40 %	68 %	58 %	NS
Interhospital transfer	123 (38.7 %)	26 (59.1 %)	97 (35.4)%	<0.01
Hospital admission	80 (25.1 %)	33 (75 %)	47 (17.2 %)	<0.0001
Hospital LOS	2.86 ± 1.13	2.84 ± 1.06	2.87 ± 1.17	NS
ICU admission	28 (35 %)	13 (39.4 %)	15 (31.2 %)	NS

### Clinical findings and FabAV Use

During the study period, 44 patients (13.8 %) received FabAV antivenin. The median dose per patient was four vials (range: 1–10; IQR: 4,6). Only five (8 %) of the 62 pediatric patients received antivenin. In 26 cases (59 %), FabAV therapy was instituted at a smaller rural hospital prior to transfer to ETMC.

A comparison of clinical findings is summarized in Table 2. Hemodynamic changes were significantly more common in patients who received FabAV (*p* < 0.05), yet these were only present in eight (18.2 %) of those patients: seven patients had tachycardia while one patient required vasopressor therapy. FabAV administration was more common after upper extremity snakebite (*p* < 0.0001) and in patients with moderate swelling (*p* < 0.01); no patients were characterized as having “severe” swelling. The presence or absence of constitutional symptoms, numbness, bruising, and erythema did not predict FabAV administration. Abnormal coagulation tests were present in 75 patients (23.6 %) with no difference between those who did and did not receive antivenin. Of these, 74 were mild (grade 1) and one was moderate (grade 2) abnormalities based on the SSS criteria. [5].

**Table 2:** Clinical findings

Bite location		Total (318)	FabAV (44)	No FabAV (274)	*p*
	Upper (%)	130 (40.9 %)	35 (80 %)	95 (35 %)	<0.0001
	Lower (%)	185 (58.2 %)	9 (20 %)	176 (64 %)	
	Torso	3 (<1 %)	0	3 (<1 %)	
Systemic symptoms					
Cardiovascular symptoms		31 (9.7 %)	8 (18.2 %)	23 (8.4 %)	<0.05
Constitutional symptoms		24 (17.3 %)	5 (11.4 %)	19 (6.9 %)	NS
Abnormal coagulation tests		75 (23.6 %)	13 (29.5 %)	62(22.6 %)	NS
Local symptoms					
Numbness		19 (6.0 %)	4 (9.1 %)	15 (5.5 %)	NS
Bruising		26 (8.2 %)	5 (11.4 %)	21 (7.7 %)	NS
Erythema		112 (35.2 %)	16 (36.4 %)	96 (35.0 %)	NS
Swelling – Degree					
	None (0)	50 (15.7 %)	1 (2.3 %)	49 (17.9 %)	<0.005
	Mild (1)	235 (73.9 %)	34 (77.3 %)	201 (73.4 %)	
	Moderate (2)	33 (10.4 %)	9 (20.5 %)	24 (8.8 %)	
	Severe (3)	0	0	0	
	Median (IQR)	1 (1,1)	1 (1,1)	1 (1,1)	
Pain – Degree					
	Mild (1)	254 (79.9 %)	38 (86.4 %)	216 (78.9 %)	NS
	Moderate (2)	49 (15.4 %)	3 (6.8 %)	46 (16.8 %)	
	Severe (3)	15 (4.7 %)	3 (6.8 %)	12 (4.4 %)	
	Median (IQR)	1 (1,1)	1 (1,1)	1 (1,1)	

The calculated snakebite severity scores compared to FabAV use are presented in Table 3. For the study population, 280 patients (88 %) had mild envenomation (SSS ≤ 3), 38 patients (12 %) had moderate envenomations (SSS 4–7) and no patients had clinical evidence of severe envenomation (SSS ≥ 8). The average calculated SSS was significantly higher in patients who received FabAV (*p* < 0.001). However, the average SSS in FabAV patients was still less than3 (2.8 ± 1.5); median 2 (range: 1–7; IQR: 2,4). Based on SSS, 70.5 % of patients given FabAV sustained only mild envenomation. Overall, SSS was poorly correlated with FabAV administration (r = 0.238; *p* < 0.00002). Further, most patients with SSS > 3 (25 of 38) did not receive FabAV. As a predictor of FabAV administration, the category “SSS >3”, was also very poorly correlated with its use (r = 0.217; *p* < 0.0001).

**Table 3:** Snakebite severity score

Snakebite severity score	Total (318)	FabAV (44)	No FabAV (274)	*p*
Mild (≤3)	280 (88.1 %)	31 (70.5 %)	249 (90.9 %)	<0.001
Moderate (4–7)	38 (22.9 %)	13 (29.5 %)	25 (9 %)	
Severe (≥8)	0	0	0	
Mean ± SD	2.1 ± 1.2	2.8 ± 1.5	2.0 ± 1.1	<0.0001
Median (IQR)	2 (1,3)	2 (2,4)	2 (1,3)	

There were no deaths. Most patients given antivenin (75 %) were admitted to the hospital; among those not receiving FabAV, 17.2 % were admitted and observed. Length of stay and intensive care unit admissions were similar among admitted patients. No patients developed compartment syndrome or required any surgical procedures after copperhead snakebite. There were no wound infections nor were there any reported or observed reactions to FabAV in the study population.

## Discussion

In the present study, we analyzed the management of copperhead snakebite at a tertiary referral center in East Texas with an emphasis on the use of FabAV and the clinical indications for its use. We report a significant variation in practice regarding FabAV administration. While most patients, even those with moderate envenomation, were managed without receiving FabAV, the majority of patients who did receive FabAV only had clinical evidence of mild envenomation. Three-fourths of patients who received FabAV were admitted to the hospital, presumably to monitor for adverse reactions to the antivenin rather than their degree of envenomation. The snakebite severity score, a validated research tool which is also often used to determine the need for antivenin therapy [2, 5], only poorly correlated with FabAV use indicating that degree of envenomation was not the primary factor driving the decision to administer antivenin after copperhead snakebite. Further, we demonstrate a low overall degree of envenomation after copperhead snakebite for a large cohort of patients.

Our findings reflect those of other authors who have concluded that snakebite from known or presumed copperhead pit vipers rarely require antivenin therapy [1, 8, 9]. While local symptoms such as swelling and bruising can be alarming after copperhead snakebite, severe envenomations with systemic manifestations such as shock, clinical bleeding from coagulopathy, respiratory failure, or death are essentially unheard of outside of an intravenous envenomation or an anaphylactic reaction to the venom (which is not treated principally with antivenin but rather also with steroids, antihistamines, etc.) [10–15].

More importantly, the present study confirms other reports stating that often little clinical evidence, or standardized guidelines, drive costly FabAV administration after copperhead snakebite [13]. While other retrospective studies have demonstrated that FabAV can limit the progression of local tissue effects after copperhead envenomation, there is currently no data to suggest that the cost and risk associated with FabAV improves long-term limb function or leads to any other measurable outcome improvements in these patients [11].

Although only 44 of 318 patients received FabAV in our series, it is likely that very few, if any, of these patients actually required antivenin therapy based on their clinical presentation as documented in the medical record. As such, it appears that these patients realized little or no benefit from receiving the drug. While certainly the FabAV itself could have prevented recipients from evolving more severe symptoms, the overall benign initial presentations suggest otherwise.

These data suggest that some physicians treat patients with FabAV independent of severity of envenomation. Whether the cause is a lack of experience or education, medico-legal fears, or a reliance on algorithms meant for rattlesnake/cottonmouth envenomations, the overuse of FabAV after copperhead snakebite must be addressed. While the risks of the medication itself are relatively low (8 % hypersensitivity and 13 % serum sickness), cost is certainly a factor: each vial has a wholesale cost of approximately $2000 and patients may be charged as much as $20,000 per vial for the antivenin alone [1, 16, 17]. With a four-vial initial dose, plus hospital admission and other costs such as interhospital transfer, medical bills can quickly mount up – all with marginal proven benefit.

Interestingly, copperhead snakebites were completely excluded from the initial clinical trials involving FabAV and all prospective data regarding outcomes is based on rattlesnake envenomations which are clearly much more dangerous [11, 13, 18]. This fact, coupled with other case series also documenting significant practice variation and questionable clinical benefit, has led to the rare decision by researchers to undertake a post-marketing double-blind placebo-controlled randomized clinical trial (RCT) comparing FabAV to placebo after copperhead envenomation which is currently enrolling patients in 14 centers [1, 8, 11, 13, 19].

While the results of the RCT currently in progress should provide much-needed guidance and will support a standardized approach with regard to FabAV use for copperhead snakebite, there does already appear to be enough retrospective data, from this and previous studies, to recommend using common sense, experience, and clinical judgment to guide an expectant approach for mild to moderate copperhead envenomations and to suggest that definitive clinical findings indicating a severe envenomation should be present before FabAV is given in most patients [1, 8, 11]. Because management of copperhead snakebite without antivenin treatment is well established as safe, only patients likely to experience a tangible clinical benefit should receive this costly drug [1, 8, 11, 20]. Automatic or standardized administration of FabAV for copperhead snakebite should be discouraged.

## Limitations

The main limitation of the present study is certainly its retrospective nature. Reliance on medical record descriptions of signs and symptoms of snakebite is inadequate to truly judge the severity of envenomation. Further, in the absence of prospective analysis, patients were not evaluated consistently and not all patients had adequate laboratory testing, especially repeat testing. An additional limitation is our analysis of a single institution’s experience creating a more narrow perspective that a more regional analysis might give. Although ETMC is a tertiary referral center, and most patients who receive FabAV are transferred in for “higher level of care services”, some venomous snakebite patients are managed at smaller local hospitals preventing their inclusion in our analysis.

## Conclusions

There is considerable variation in FabAV administration after copperhead snakebite in East Texas – which reflects the national experience. While current standardized scoring guidelines (SSS > 3) for antivenin administration are likely useful for rattlesnake and cottonmouth envenomations, these criteria may be too liberal to guide treatment after copperhead snakebite. Given the cost of treatment, and the lack of proven clinical benefit, we suggest, for patients with copperhead snakebite, that consideration be given to withholding FabAV for those without clinical evidence of severe envenomation. The RCT, currently in progress, is clearly needed to clarify the treatment algorithm and provide concrete guidelines for physicians caring for copperhead snakebite victims.

### Ethics approval

Approval was obtained, including waiver of consent, from the East Texas Medical Center (ETMC) institutional review board for this retrospective medical record query (IRB protocol #674).

## Abbreviations

SSS: snakebite severity score
FabAV: Crotalidae polyvalent immune Fab (ovine)
